# Isomaltooligosaccharides Production Using α-Glucosidase Activity from *Zalaria* sp. Him3, a Fructooligosaccharides-Producing Yeast

**DOI:** 10.1007/s00284-025-04392-x

**Published:** 2025-08-01

**Authors:** Haruki Matsuya, Mayumi Maeda, Kenji Maehashi, Jun Yoshikawa

**Affiliations:** 1 Department of Fermentation Science and Technology, Graduate School of Applied Bioscience, Tokyo University of Agriculture, 1-1-1 Sakuragaoka, Setagaya-Ku, Tokyo, 156-8502 Japan; 2 Department of Fermentation Science, Faculty of Applied Bioscience, Tokyo University of Agriculture, 1-1-1 Sakuragaoka, Setagaya-Ku, Tokyo, 156-8502 Japan

## Abstract

In this study, we aimed to develop isomaltooligosaccharides (IMO) production as a novel industrial application of *Zalaria* sp. Him3, fructooligosaccharides (FOS)-producing yeast. A utilization test of carbon sources by *Zalaria* sp. Him3 was performed using API 50CH. Subsequently, the strain was cultivated in maltose medium, and the culture supernatant was used as α-glucosidase (AGase). AGase activity was evaluated by determining the amount of *p*-nitrophenol (PNP) derived from PNP-α-1,4-glucoside. IMO production was measured at 30 °C in the reaction mixture containing 280 g/L maltose and 0.25 U/mL AGase. *Zalaria* sp. Him3 highly utilized glucose, mannose, maltose, sucrose, and trehalose. When the strain was incubated with 150 g/L maltose for 48 h, IMO (isomaltose, panose, and isomaltotriose) were confirmed in the culture supernatant and AGase activity was 0.0165 U/mL. During IMO production using AGase in the culture supernatant, approximately 98% of initial maltose (279 g/L) was degraded for 72 h. The maximum IMO concentration was 138 g/L after 12 h of reaction. Thus, the yield of IMO production by AGase was 49.5% of the initial maltose. *Zalaria* sp. Him3, which is the FOS-producing yeast, can also produce IMO and serves as an industrially promising enzyme resource for the production of multiple types of oligosaccharides.

## Introduction

Isomaltooligosaccharides (IMO), such as isomaltose, panose, and isomaltotriose, mainly consist of glucosyl oligosaccharides linked by α−1,6-glycosidic linkages. IMO have shown therapeutic potential for gastrointestinal and metabolic diseases, including obesity, diabetes, and inflammatory bowel disease, by effectively stimulating the growth of beneficial gut bacteria as prebiotics [1]. Therefore, oligosaccharides are considerably promised as one of the functional sugars in the food industry. IMO are naturally found in honey and fermented foods, such as sake, miso, and soy sauce [2–5]. Industrially, IMO have been produced via transglucosyl activity of α-glucosidase (AGase), the commercial enzyme preparation derived from *Aspergillus niger* [6–8].

*Zalaria obscura*, a black yeast isolated from house dust was proposed as a new genus and species [9]. In addition, *Zalaria* spp. were isolated from various sources, including blackened wooden artwork, clean room, and dried sweet potato in Italy, North America, and Japan, respectively [10–12]. Recently, *Zalaria* sp. Him3 was reported to have β-fructofuranosidase (FFase) activity for fructooligosaccharides (FOS) production, suggesting that this yeast species can be effectively applied in the bioindustry [12]. However, only a few studies related to *Zalaria* have been reported, and its physiological property remains poorly understood.

In this study, we aimed to develop the novel application of *Zalaria* sp. Him3. A utilization test of carbon sources and oligosaccharides production were performed on this yeast strain, and the AGase activity in the culture supernatant was measured when the strain was grown in the maltose medium. Moreover, IMO production using AGase was performed.

## Materials and Methods

### Strain

*Zalaria* sp. Him3 was isolated from dried sweet potato as previously reported [12]. The strain was maintained on a storage agar medium (6.7 g/L Yeast nitrogen base [BD, NJ, USA], 10 g/L glucose, 1 g/L yeast extract, and 15 g/L agar) and stored at 4 °C.

### Culture Media and Cultivation

For the seed culture, a small amount of colony was inoculated into a test tube containing 3 mL of a seed medium (20 g/L maltose, 10 g/L yeast extract, and 5 g/L polypeptone) and incubated at 30 °C for 24 h with reciprocal shaking at 180 spm. The seed broth (0.2 mL) was inoculated into a 100-mL Erlenmeyer flask containing 20 mL of a culture medium (various concentrations of maltose or sucrose, 20 g/L yeast extract, and 5 g/L polypeptone) and incubated at 30 °C for 24–72 h with rotary shaking at 180 rpm. The OD_660_ was measured as an indicator of cell growth.

### Utilization of Carbon Source by *Zalaria* sp. Him3

A utilization test was performed using API 50CH (bioMérieux S.A., Métropole de Lyon, France). The culture broth of *Zalaria* sp. Him3 was suspended in 10 mL of API 50CHB/E medium (bioMérieux S.A.) with an OD_660_ of 1. The suspension (0.18 mL) was inoculated into each tube and incubated at 30 °C for 72 h. Three days later, the color of the medium was compared with that of the control tube.

### AGase Preparation and Enzyme Assay

Strain Him3 was grown in the culture medium for 48 and 72 h and centrifugated at 16,000 × *g* for 3 min at 4 °C. The culture supernatant was collected as the AGase preparation, and the enzyme activity was measured by determining the amount of *p*-nitrophenol (PNP) derived from PNP-α−1,4-glucoside (PNPG). The reaction mixture contained 0.1 mL of AGase and 0.6 mL of 6 mM PNPG dissolved in a 50 mM sodium acetate buffer (pH 5.0). After incubation at 30 °C for 30 min, the reaction was stopped by adding 0.3 mL of 0.2 M Na_2_CO_3_. The PNP absorbance in the reaction mixture was measured at 400 nm. One unit (U) of the AGase activity was defined as the amount of enzyme that released 1 µmol of PNP per min.

### Thin Layer Chromatography (TLC) Analysis

The culture supernatant (1 µL) was applied to TLC and developed using n-butanol/ethanol/water (5:3:2, v/v/v) as the eluent. Spots (a positive reaction) were detected by heating after spraying with 30% sulfuric acid.

### High-Performance Liquid Chromatography (HPLC) Analysis

The sugar concentrations in the culture supernatant and enzyme reactant were determined using HPLC. The analysis conditions were as follows: column, HILICpak VG-50 4E (4.6 mm × 250 mm; Resonac, Tokyo, Japan); mobile phase, acetonitrile–water (75:25 (v/v)); flow rate, 0.6 mL/min; temperature, 40 °C; detector, refractive index detector. The sugar concentrations were determined using standard curves prepared for each sugar.

### IMO Production Using AGase

AGase was prepared as described above and concentrated by ultrafiltration (Amicon Ultra-15, 10,000 Da cut off; Merck, Darmstadt, Germany). IMO production was performed in 10 mL of reaction mixture consisting of 280 g/L maltose dissolved in 100 mM sodium acetate buffer (pH 5.0) and 0.25 U/mL AGase at 30 °C. The sample was sequentially collected, and the enzyme reaction was stopped by heating at 100 °C for 10 min. The reaction products were determined using HPLC as described above.

## Results and Discussion

### Utilization of Carbon Sources by *Zalaria* sp. Him3

Since glucose and sucrose have been confirmed as the carbon sources that *Zalaria* sp. Him3 can utilize [12], other carbon sources were evaluated using API 50CH. The utilized carbon sources in this study were shown in Table 1. Highly positive reactions were observed for mannose, maltose, trehalose, sucrose, and glucose, whereas weak responses were observed for erythritol, xylose, fructose, galactose, and cellobiose. Several carbohydrates (arabinose, ribose, lactose, melibiose, and starch) and sugar alcohols (glycerol, xylitol, mannitol, and sorbitol) were not utilized. *Z. obscura* utilized glucose, fructose, lactose, sucrose, xylose, glycerol, and mannitol for growth [13]. These results indicated that *Zalaria* sp. Him3 and *Z. obscura* utilize different carbon sources, and that these strains are physiologically distinct species.

**Table 1:** Utilization of carbon sources by *Zalaria* sp. Him3

Carbon sources	Utilization
Erythritol Xylose Fructose Galactose Glucose Mannose Cellobiose Maltose Sucrose Trehalose	+ + + + + + + + + + + + + + +

The test was performed using API 50CH, with the evaluation based on the results of two independent experiments. The color of the control medium was red. The medium color associated with a pH reduction after incubation was used to determine the utilization level of each carbon source (vermilion, +; yellow, + +)

### Confirmation of Oligosaccharides Production by *Zalaria* sp. Him3

**Fig. 1:**
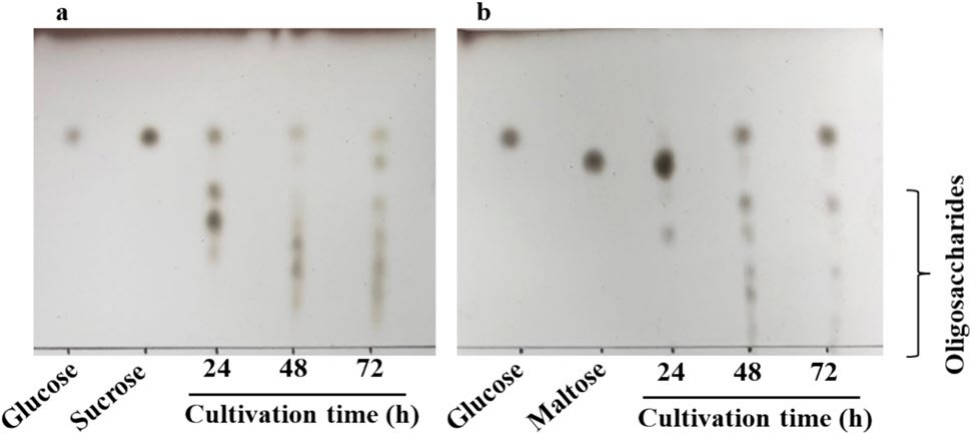
Thin-layer chromatography analysis of the culture supernatant from *Zalaria* sp. Him3 cultivated with sucrose (**a**) and maltose (**b**) as a carbon source. Glucose, sucrose, and maltose are standard samples. The spots in the lower area indicate oligosaccharides with a higher degree of polymerization

**Fig. 2:**
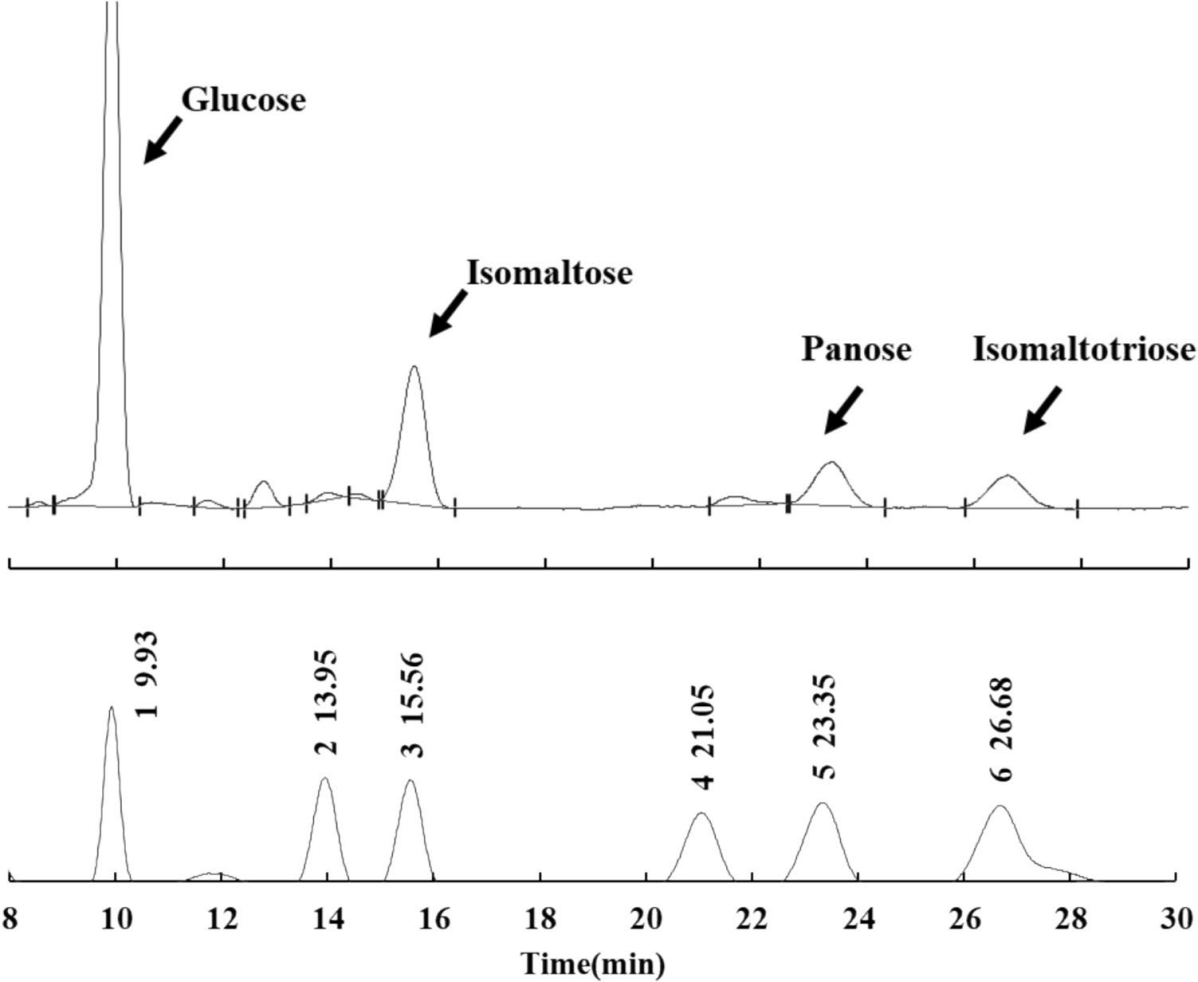
HPLC analysis of the culture supernatant from *Zalaria* sp. Him3 cultivated with maltose (upper) and sugar standards (lower). The standard peaks from 1 to 6 are glucose, maltose, isomaltose, maltotriose, panose, and isomaltotriose, respectively

As described above, strain Him 3 could utilize maltose as the carbon source. IMO are known to be generated from maltose by the transglucosylating activity of AGase [7, 8]. Thus, IMO production from maltose by *Zalaria* sp. Him3 was confirmed in the culture broth. Oligosaccharide production was determined using TLC when the strain was grown in the culture medium containing 150 g/L sucrose and maltose. In the sucrose culture, spots with a higher degree of polymerization were detected in the lower area of TLC (Fig. 1a). These spots were suggested to be FOS because the strain can produce the oligosaccharides from sucrose [12]. Similarly, oligosaccharide spots in the maltose culture were generated after 24 h (Fig. 1b). However, it was not clear whether these products were IMO, which have a physiological function. The oligosaccharides in the culture supernatant were compared with standard IMO using HPLC analysis. The culture supernatant was analyzed after 48 h, and the detected peaks were consistent with those of glucose, isomaltose, panose, and isomaltotriose (Fig. 2). Based on these results, it was concluded that *Zalaria* sp. Him3 produced IMO from maltose.

### AGase Production by *Zalaria* sp. Him3

**Fig. 3:**
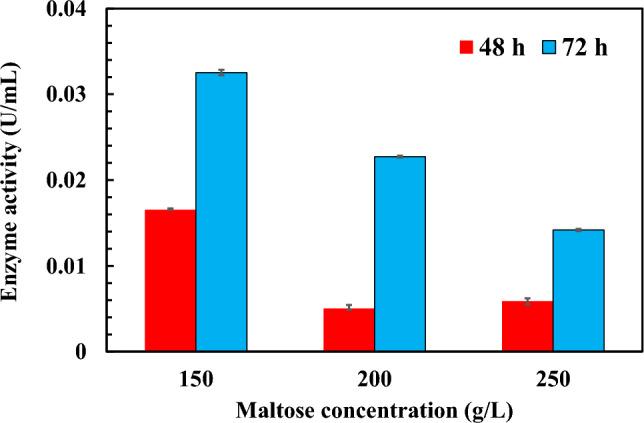
AGase activities of *Zalaria* sp. Him3 cultivated with various maltose concentrations for 48 h (red bars) and 72 h (blue bars). The data are shown as the means ± SD of three independent 274 experiments (Color figure online)

Strain Him3 was grown in a culture medium containing 150–250 g/L maltose, and AGase activity related to IMO production was measured using the culture supernatant after maltose cultivation for 48 and 72 h (Fig. 3). AGase production was the highest under 150 g/L maltose condition, and the activity was 0.0165 and 0.0325 U/mL after 48 and 72 h, respectively. The OD_660_ of broth cultivated in 250 g/L maltose medium was lower than that in 150 g/L maltose (data not shown). A decrease in the enzyme activity with maltose concentration may be influenced by cell density during the growth. As shown in Fig. 1b, owing to the decrease in oligosaccharide spots after 72 h, the enzymes would exert not only transglucosylating activity but also hydrolytic activity. Thus, the culture supernatant under the 150 g/L maltose condition for 48 h was thought to be a suitable AGase resource for IMO production.

### IMO Production Using AGase from *Zalaria* sp. Him3

**Fig. 4:**
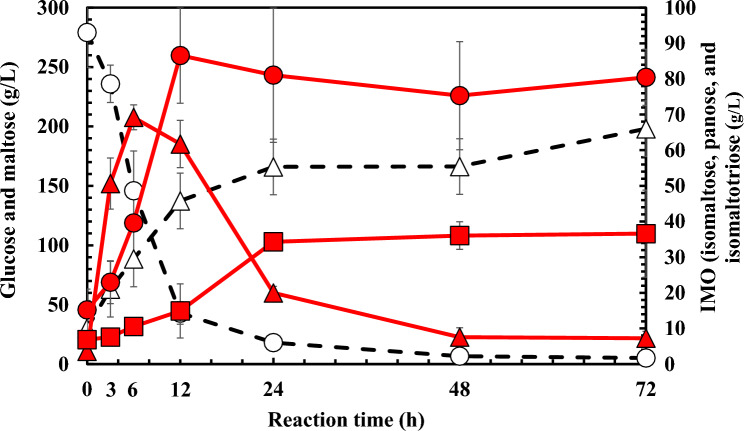
IMO production using AGase from *Zalaria* sp. Him3. Maltose, open circles; isomaltose, close circles; glucose, open triangles; panose, close triangles; isomaltotriose, close squares. The data are shown as the means ± SD of three independent experiments

AGase prepared above was reacted with a high concentration of maltose as a substrate for IMO production. As shown in Fig. 4, initial maltose (279 g/L) was degraded by AGase, and approximately 98% of maltose was consumed after the reaction for 72 h. The maximum IMO concentration, a sum of isomaltose, panose, and isomaltotriose minus the initial values, was 138 g/L for 12 h. Thus, the IMO yield to initial maltose generated by AGase was calculated to be 49.5%. In the early stage of IMO production, panose was produced by the transglycosylation of glucose derived from maltose with the other maltose as an acceptor, and remaining glucose was liberated. Isomaltose and isomaltotriose were probably produced using liberated glucose as the acceptor in the transglucosylation reaction, while panose would be consumed as a glucose donor after the maltose decrease. However, in IMO production by *Aureobasidium pullulans* and *Aspergillus niger*, panose was hardly degraded [7, 14–16]. In contrast, the enzymes from *Aspergillus neoniger* and *Thermoanaerobacter thermocopriae* synthesized panose faster than isomaltose, and the trisaccharide was decreased [17, 18]. The degradation of panose should not be affected by the contaminant enzyme because AGases from *A. neoniger* and *T. thermocopriae* were purified after heterologous expression. Thus, the reaction mechanism of these AGases may be two types of transglucosylation, either panose-dependent or panose-independent as the substrate. The sugar component of oligosaccharides differed in the reaction period, and it may be important for the food industry to provide various IMO with different physical properties depending on the application. In a future study, AGase for IMO production will be identified in *Zalaria* sp. Him3, and its enzymatic properties will be clarified.

## Conclusion

In this study, the utilization of various carbon sources in *Zalaria* sp. Him3 was revealed. Strain Him3 utilized erythritol, xylose, fructose, galactose, and cellobiose but did not seem to metabolize lactose, glycerol, and mannitol. Its utilization of carbon sources was different from that of *Z. obscura*. In addition, IMO production using AGase from strain Him3 was demonstrated, and the maximum IMO concentration and yield to initial maltose as the substrate were 138 g/L and 49.3%, respectively. This is the first study to report the detection of AGase activity and IMO production using *Zalaria* spp. These results show that *Zalaria* sp. Him3 can express the enzymes for IMO and FOS production by using maltose and sucrose as the carbon source, respectively. Therefore, *Zalaria* sp. Him3 is expected to be used as a promising enzyme resource for the industrial production of multiple types of oligosaccharides.

## Data Availability

The datasets generated during and/or analyzed during the current study are available from the corresponding author on reasonable request.
